# Green Management Strategies for *Cochliomyia hominivorax* Myiasis: Potential of Essential Oils and Nanodelivery Systems

**DOI:** 10.3390/biology15181654

**Published:** 2026-09-18

**Authors:** Romario García-Ponce, José P. Villarreal-Villarreal, Jesús J. Hernández Escareño, Nancy N. Espinosa-Carranza, Michel Stéphane Heya, Karla L. Silva-Martínez, José A. Rosado-Aguilar, Gustavo Hernández-Vidal

**Affiliations:** 1 Facultad de Medicina Veterinaria y Zootecnia, Universidad Autónoma de Nuevo León, Gral. Escobedo 66054, NL, Mexico; romario.garciapo@uanl.edu.mx (R.G.-P.); pablo.villarrealvl@uanl.edu.mx (J.P.V.-V.); jesus.hernandezec@uanl.edu.mx (J.J.H.E.); 2 Facultad de Ciencias Biológicas, Universidad Autónoma de Nuevo León, San Nicolás de los Garza 66455, NL, Mexico; nancy.espinosacrr@uanl.edu.mx; 3 Facultad de Salud Publica y Nutrición, Universidad Autónoma de Nuevo León, Monterrey 64460, NL, Mexico; michel.heyax@uanl.edu.mx; 4 Instituto Tecnológico Superior de Tantoyuca, Tecnológico Nacional de México, Tantoyuca 92100, VER, Mexico; karla.silva@itsta.edu.mx; 5 Facultad de Medicina Veterinaria y Zootecnia, Universidad Autónoma de Yucatan, Mérida 97000, YUC, Mexico

**Keywords:** bioactive compounds, nanoinsecticide, myiasis, nanoemulsions, polymeric nanoparticles

## Abstract

*Cochliomyia hominivorax*, the main etiological agent of traumatic myiasis in the New World, continues to have significant health and economic impacts due to its effects on animal health, livestock production and public health in affected regions. Although conventional control strategies, including the Sterile Insect Technique and chemical insecticides, have substantially reduced its distribution, concerns regarding insecticide resistance, environmental impacts, and recent re-emergence events underscore the need for sustainable alternative control approaches. This review summarizes current advances in the use of EOs and plant-derived bioactive compounds for the management of myiasis-causing flies, with particular emphasis on *C. hominivorax*, and discusses the emerging role of nanocarrier-based delivery systems in enhancing their biological performance. EOs and their major constituents exhibit larvicidal, ovicidal, adulticidal, repellent, and oviposition-deterrent effects through multiple mechanisms that disrupt essential physiological and developmental processes in insects. Several EOs have shown promising activity against *C. hominivorax* larvae; however, their practical application is often limited by their high volatility, low aqueous solubility, and physicochemical instability. Nanodelivery systems, including nanoemulsions, polymeric nanoparticles, and solid lipid nanoparticles, offer effective strategies to improve the stability, bioavailability, controlled release, and overall efficacy of plant-derived bioactive compounds. Current evidence indicates that nanodelivery systems enhance the stability and insecticidal efficacy of EOs while reducing the concentrations required to achieve biological activity, supporting their potential for the prevention and treatment of *C. hominivorax* myiasis. Nevertheless, further in vivo and field studies are required to validate their practical application.

## Simple Summary

Myiasis is a disease caused by fly larvae that feed on the living tissue of animals and, occasionally, humans, causing severe wounds, reduced animal welfare, and significant economic losses. Current control relies mainly on chemical insecticides, whose extensive use has promoted insecticide resistance and environmental concerns. Plant essential oils (EOs) are promising natural alternatives because of their insecticidal and repellent properties, but their instability limits practical application. Nanotechnology-based delivery systems can protect these compounds, improving their stability, prolonging their activity, and enhancing their biological efficacy. This review summarizes current knowledge on essential oils and nanocarrier systems for the control of myiasis-causing flies, with emphasis on the New World screwworm. Although further studies are needed to validate these approaches under field conditions, they represent a promising and more sustainable strategy for myiasis prevention and management in veterinary and human health.

## 1. Introduction

The New World screwworm, *Cochliomyia hominivorax*, is an obligate parasitic fly of considerable veterinary and public health importance, affecting a broad range of mammalian hosts, including livestock, wildlife, companion animals, and humans [1,2,3]. Infestation by this species results in traumatic myiasis, a condition characterized by larval invasion and feeding on living or necrotic vertebrate tissues, leading to extensive tissue destruction. Such lesions increase host susceptibility to secondary bacterial infections, toxemia, and severe physiological stress [4]. Consequently, *C. hominivorax* constitutes a major threat to animal health and welfare, with substantial consequences for livestock production throughout endemic regions of the Americas. In addition, infestations are associated with economic losses and have major implications for both animal and human health [3,5,6].

The economic impact associated with myiasis caused by *C. hominivorax* is substantial. In several South American countries, the costs associated with the prevention and treatment of these infestations reach millions of dollars annually. In Uruguay, annual expenditures have been estimated at between USD 40 and 52.2 million [7], whereas in Argentina, economic losses range from USD 55 to 60 million, considering only five provinces [8]. Similarly, the economic burden on the Brazilian livestock sector is estimated at approximately USD 340 million per year [9].

In North America, particularly in Mexico, *C. hominivorax* was eradicated in 1991 through a transnational federal program. The program was based primarily on the mass release of sterile males and was complemented by additional control measures. However, since November 2024, the reappearance of this species has been reported in several Mexican states [10]. This renewed occurrence demonstrates the continuing risk posed by *C. hominivorax* in the region and reinforces the need for effective and sustainable control strategies.

Historically, New World screwworm control has relied on integrated management strategies that include epidemiological surveillance, the Sterile Insect Technique, and the use of chemical insecticides or systemic antiparasitic drugs [3,11]. While these approaches have successfully eradicated *C. hominivorax* from several regions, including the United States and parts of Central America, the species remains endemic in numerous tropical areas where climatic and ecological conditions favor its establishment and spread [12]. Moreover, anthropogenic activities, particularly the movement and commercial trade of livestock and companion animals, have been recognized as major drivers of its geographic spread, facilitating the reintroduction of the parasite into previously eradicated areas [5].

However, the emergence of resistance to insecticides and systemic antiparasitic compounds poses a significant challenge to the long-term effectiveness of current control programs [13,14,15,16]. Such resistance may compromise treatment efficacy, increase management costs, and intensify economic losses in livestock production systems. Furthermore, the widespread use of chemical insecticides has raised environmental and public health concerns owing to their detrimental effects on non-target organisms, environmental persistence, contamination, and potential risks to animal and human health [17,18,19]. Together, these challenges underscore the need to develop safer, more sustainable, and environmentally friendly alternatives for the management of *C. hominivorax* infestations.

In recent years, plant-derived bioactive compounds have attracted increasing attention as potential alternatives for the control of insects and parasites of medical, veterinary, and agricultural importance. Among these compounds, terpenoids, phenolic compounds, and alkaloids have demonstrated insecticidal, larvicidal, repellent, adulticidal, antifeedant, and growth-regulating activities against a wide range of arthropod species [20,21,22,23]. In particular, EOs, which are composed primarily of monoterpenes, sesquiterpenes, and phenylpropanoids, have demonstrated the ability to interfere with essential physiological processes in insects, including neuromuscular dysfunction, acetylcholinesterase inhibition, tissue damage, induction of oxidative stress, and disruptions in development and reproduction [24,25,26,27].

Moreover, their biological activity has been shown to depend not only on their major constituents but also on complex and synergistic interactions among major and minor metabolites, which may significantly enhance their insecticidal efficacy [28]. In addition, these compounds are regarded as environmentally friendly alternatives to conventional synthetic insecticides because of their lower environmental persistence and reduced potential for resistance development, a characteristic attributed to the chemical complexity of their natural mixtures [29,30,31,32]. Nevertheless, their practical application remains limited by several factors, including high volatility, poor water solubility, low stability under environmental conditions, and rapid degradation following application, all of which can compromise their effectiveness under field conditions [33,34,35,36].

Nanotechnology has emerged as a promising approach to overcome the major limitations associated with the pharmaceutical development and practical application of EOs for the control of myiasis-causing flies. Encapsulation of EOs and their bioactive constituents into nanocarrier systems enhances their physicochemical stability by protecting them from volatilization, oxidation, and environmental degradation, while facilitating the incorporation of poorly water-soluble bioactive compounds into aqueous formulations and enabling controlled release [35,37,38,39,40]. These properties may optimize the pharmacological performance of EOs by increasing their biological efficacy and reducing the concentrations required to achieve insecticidal effects. Among the nanocarrier platforms investigated for botanical compounds, nanoemulsions, polymeric nanoparticles, liposomes, and lipid nanoparticles have demonstrated considerable potential to improve encapsulation efficiency, stability, and the delivery of bioactive compounds [36,41,42,43,44,45]. Furthermore, nanoformulated botanical insecticides have shown enhanced efficacy against agricultural pests and dipteran vectors, supporting their potential as sustainable alternatives for the prevention and control of *C. hominivorax* myiasis [46,47,48,49].

Despite the increasing interest in botanical insecticides and nanotechnology-based delivery systems, information regarding their application for the control of myiasis-causing flies remains limited and fragmented. In particular, no comprehensive review has integrated the current evidence on the insecticidal potential of EOs and nanocarrier technologies for the management of *Cochliomyia hominivorax*. Therefore, the aim of this review is to critically summarize the available evidence on the biological activity of EOs against *C. hominivorax* and other myiasis-causing dipteran species, as well as to discuss the potential of nanocarrier-based delivery systems to overcome the limitations associated with plant-derived bioactive compounds. Furthermore, this review identifies current management strategies and future research perspectives to support the development of safer, more effective, and environmentally sustainable strategies for the prevention and control of traumatic myiasis.

## 2. Literature Search Strategy

This review was conducted following the Preferred Reporting Items for Systematic Reviews and Meta-Analyses (PRISMA 2020) guidelines [50]. A systematic literature search was performed in the Scopus, Web of Science, PubMed, and Google Scholar databases, including all records available from inception to May 2026. The search strategy combined the terms “*Cochliomyia hominivorax*”, “New World screwworm”, “myiasis”, “blowflies”, “essential oils”, “plant-derived bioactive compounds”, “botanical insecticides”, “nanoemulsions”, “polymeric nanoparticles”, “solid lipid nanoparticles”, and “nanocarriers” using Boolean operators (AND, OR).

Because of the limited number of experimental studies evaluating EOs and nanocarrier systems against *C. hominivorax*, the search was expanded to include studies involving other dipteran species responsible for traumatic myiasis (*Lucilia* spp., *Chrysomya* spp., *Calliphora* spp., *Cochliomyia macellaria*, and *Cephalopina titillator*). This approach provided complementary evidence on the insecticidal activity of EOs and the potential application of nanocarrier systems for myiasis control.

Original peer-reviewed in vitro studies evaluating the biological activity of EOs or plant-derived bioactive compounds against myiasis-causing flies were included. Review articles, conference abstracts, editorials, duplicate records, and studies lacking biological evaluation were excluded.

**Figure 1:**
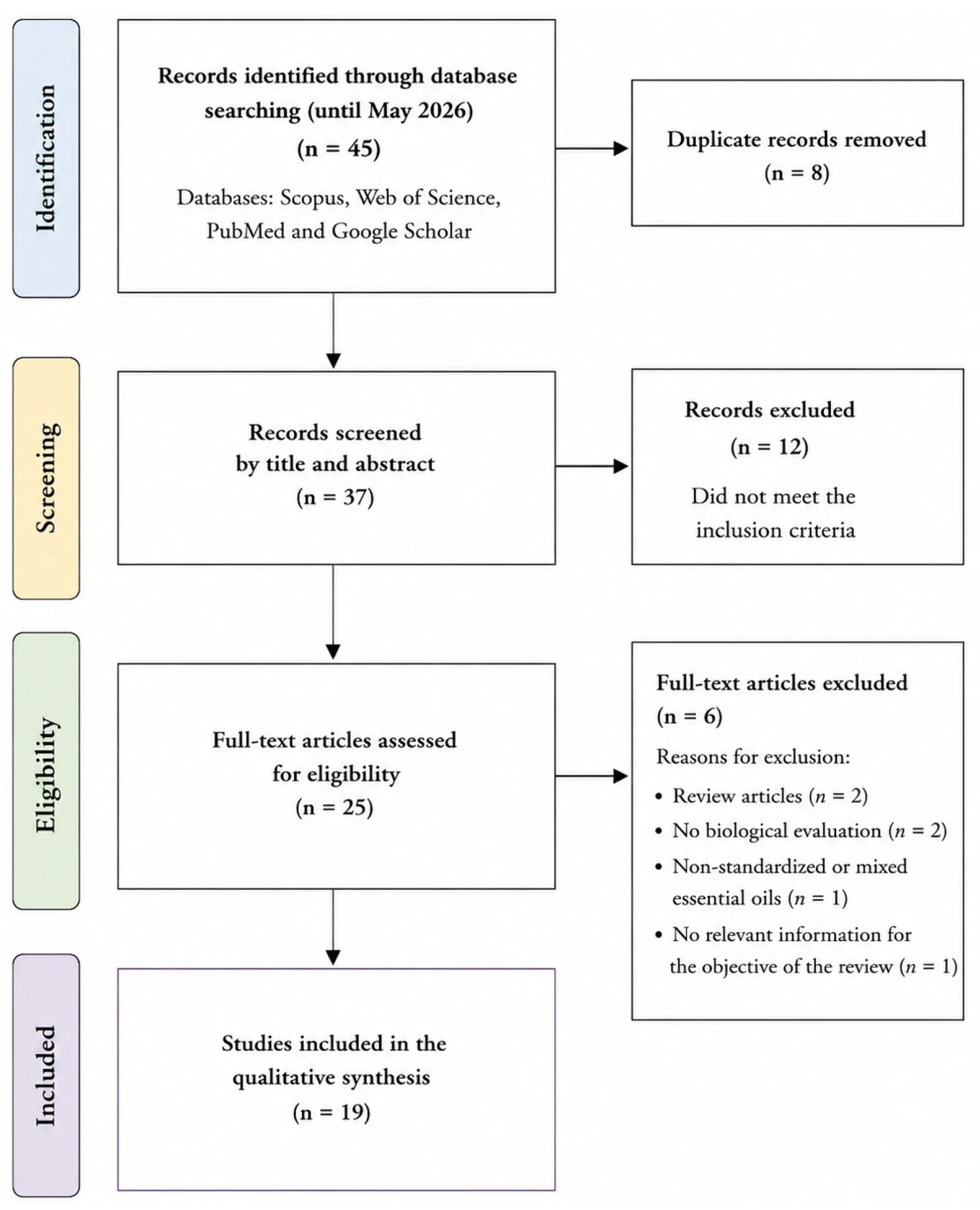
Flow diagram of the literature search and study selection process.

The literature search identified 45 records, of which 19 met the eligibility criteria and were included in the qualitative synthesis (Figure 1). Data extracted from each study included the plant species evaluated, major chemical constituents, target species, bioassays performed, and the main biological outcomes, including mortality, repellency, oviposition inhibition, and the median lethal concentration (LC_50_) and 90% lethal concentration (LC_90_). Due to the limited evidence available for *C. hominivorax* and the methodological heterogeneity among the included studies, a meta-analysis was not feasible. Therefore, the evidence was synthesized using a qualitative and comparative approach.

## 3. Biology and Pathogenesis of *Cochliomyia hominivorax*

The term “New World screwworm” (NWS) commonly refers to the larval stages of *C. hominivorax*, which are responsible for causing traumatic myiasis in animals and humans [51]. This common name originates from the characteristic behavior of the larvae, which penetrate deeply into living tissues through pre-existing wounds, burrowing into the host and creating tunnels or pockets as they feed. As a result, infestations can lead to extensive tissue destruction, secondary infections, and severe health complications if left untreated.

The species was first described by the French physician Charles Coquerel in 1858 from specimens collected on Devil’s Island in French Guiana. It was originally named *Lucilia hominivorax*, a designation that literally means “man-eating fly”, reflecting the ability of its larvae to feed on living tissues [51].

### 3.1. Biological Cycle of C. hominivorax

**Figure 2:**
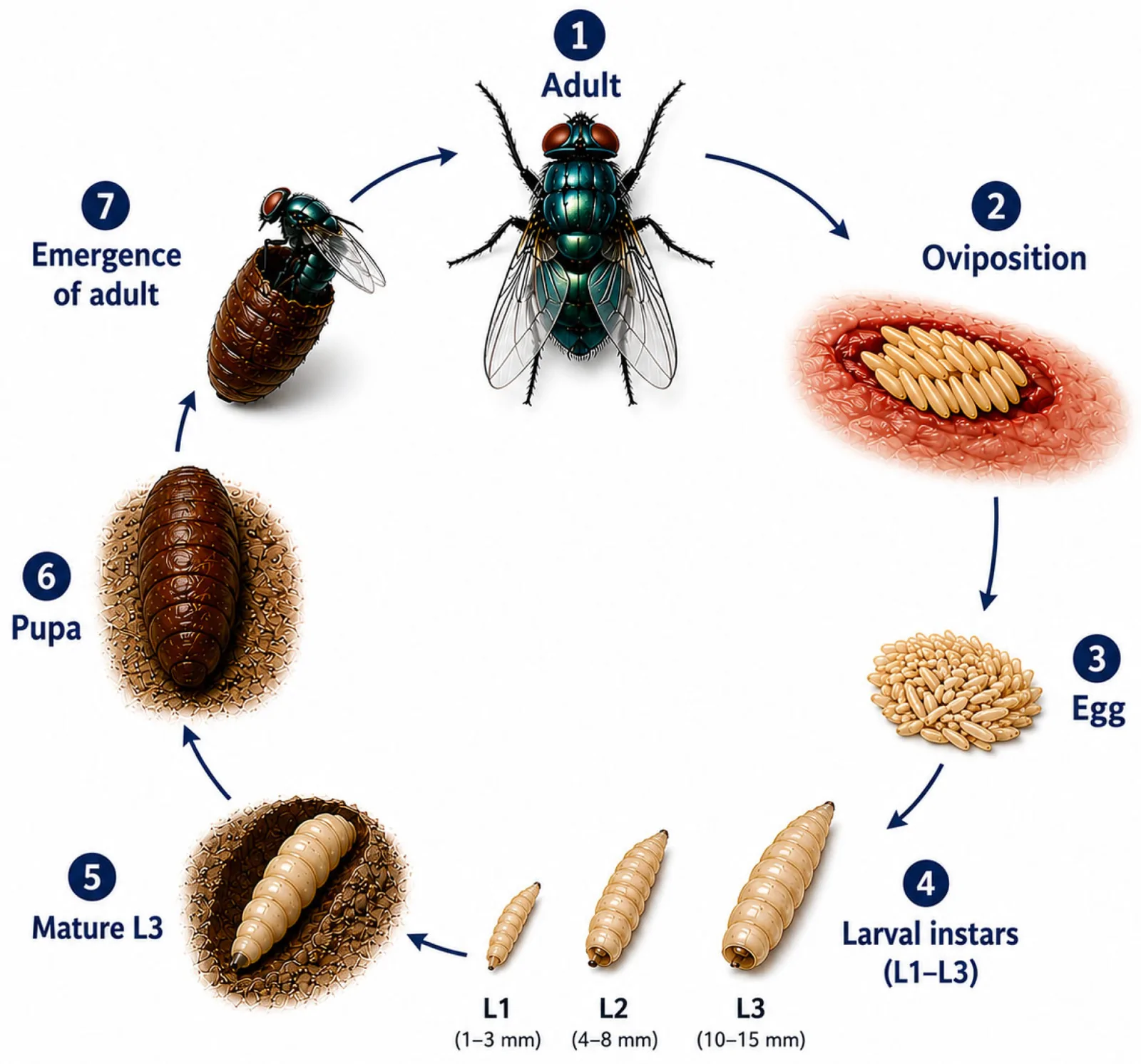
Life cycle of *Cochliomyia hominivorax*.

The life cycle of *C. hominivorax* comprises a parasitic phase, including the egg and three larval instars (L1–L3), and a free-living phase consisting of the pupal and adult stages. A schematic representation of the life cycle is shown in Figure 2. Under favorable environmental conditions (approximately 22 °C), development from egg to sexually mature adult is completed within 3–4 weeks [52]. The parasitic phase typically lasts between 4 and 8 days, whereas the duration of the pupal stage varies considerably depending on environmental conditions, particularly temperature [53].

Adult females generally mate once and, 7–9 days after emergence, oviposit egg masses containing approximately 343 eggs (up to 490 eggs) on the margins of pre-existing wounds caused by natural injuries or livestock management practices. Oviposition may also occur in natural body openings and mucosal surfaces, where gravid females are strongly attracted to damaged tissues [54,55]. Eggs hatch within 12–24 h, and the larvae immediately begin feeding on living tissue, penetrating deeply into the wound and developing through three larval instars over 4–8 days [52,54,55]. Unlike facultative myiasis-producing flies, *C. hominivorax* is an obligate parasite whose larvae cause extensive tissue destruction and frequently promote secondary bacterial infections, which may further attract gravid females and aggravate the infestation [55].

After completing development, mature third-instar larvae leave the host and pupate in the soil. Adult emergence occurs after 7–54 days, depending on environmental conditions, completing the life cycle (Figure 2) [52,55].

### 3.2. Pathogenesis of Myiasis

The larvae of *C. hominivorax* cause traumatic myiasis by feeding on the skin and underlying living tissues of their hosts, resulting in severe lesions that may become life-threatening if left untreated. The early stages of infestation are characterized by subtle larval movement within the wound, followed by rapid lesion enlargement as the larvae penetrate deeper into the surrounding tissues. Their continuous feeding activity, facilitated by robust mouth hooks and proteolytic secretions, causes extensive tissue destruction, hemorrhage, and serosanguineous exudation [56]. Infested wounds frequently develop a characteristic odor associated with tissue degradation and secondary bacterial colonization, which can further attract gravid females and stimulate additional oviposition, thereby exacerbating the infestation [53].

The pathogenesis of *C. hominivorax* infestation is characterized by the obligatory consumption of living tissue, a characteristic that differentiates this species from many other myiasis-causing flies. Progressive tissue invasion can affect not only the skin but also deeper anatomical structures, including muscle, cartilage, and bone. The resulting lesions are painful, inflammatory, and highly susceptible to secondary bacterial infections. In animals, infestations commonly occur in neonatal umbilical wounds, surgical wounds, tick bite sites, and other traumatic lesions, whereas in humans they may involve cutaneous wounds as well as nasal, auricular, oral, or ocular cavities. Without prompt treatment, infestations may progress to severe tissue destruction, systemic infection, septicemia, and, in extreme cases, death [12]. Beyond the direct pathological effects on the host, myiasis caused by *C. hominivorax* is associated with significant reductions in animal welfare and productivity. Affected animals frequently exhibit pain, irritation, reduced feed intake, weight loss, and behavioral alterations, while severe infestations may lead to substantial economic losses and increased mortality rates in livestock populations [7].

## 4. Conventional Treatments for Myiasis

The treatment of myiasis caused by *C. hominivorax* is primarily based on the elimination of larvae, wound management, and the prevention of secondary infestations. Standard therapeutic approaches include thorough wound cleaning, mechanical removal of larvae when necessary, and the application of topical larvicidal agents to eliminate remaining immature stages and promote tissue healing [57]. In severe infestations, treatment is often complemented with systemic antiparasitic drugs, anti-inflammatory agents, and supportive care to reduce tissue damage and improve animal welfare.

Several chemical compounds have been used for the treatment and prevention of screwworm infestations, including organophosphates, pyrethroids, spinosyns, and macrocyclic lactones such as ivermectin and doramectin [58]. In livestock production systems, these compounds may be applied directly to wounds or administered systemically to provide both therapeutic and prophylactic protection. Macrocyclic lactones have become widely adopted due to their endectocidal activity and ease of administration [59].

Preventive strategies frequently involve the application of insecticidal formulations to wounds resulting from routine management procedures, such as castration, dehorning, branding, and shearing. These treatments aim to reduce the attraction of gravid females, prevent oviposition, and minimize the establishment of larval infestations. In addition, insecticide sprays, dips, and pour-on formulations have been employed to reduce fly populations and lower the risk of myiasis in susceptible animals [16].

Despite their effectiveness, the extensive and prolonged use of chemical insecticides has raised concerns regarding environmental contamination, chemical residues, and the emergence of resistant populations. In South America, *C. hominivorax* has historically been controlled through the intensive use of organophosphates; however, continuous exposure to these compounds has contributed to the selection of resistant strains [60]. Similarly, reports of reduced susceptibility to macrocyclic lactones, particularly doramectin and ivermectin, have highlighted the growing challenge of antiparasitic resistance in screwworm control programs [16].

Antiparasitic resistance arises when resistant individuals survive treatment and subsequently transmit resistance-associated traits to their offspring. The development of resistance is influenced by multiple factors, including parasite biology, host immunity, treatment frequency, drug properties, and livestock management practices [61]. Consequently, the increasing occurrence of resistance, together with concerns related to environmental sustainability and treatment efficacy, underscores the need to explore alternative and complementary control strategies for the management of *C. hominivorax* myiasis.

## 5. Essential Oils as Botanical Insecticides

EOs are hydrophobic natural products derived from plant secondary metabolism and are synthesized in specialized structures, including glandular trichomes, epidermal and secretory cells, and internal secretory cavities. These oils are obtained from different plant organs and comprise complex mixtures of low-molecular-weight volatile organic compounds. Their chemical composition is dominated by terpene hydrocarbons, particularly monoterpenes and sesquiterpenes, together with a diverse array of oxygenated derivatives such as alcohols, aldehydes, ketones, esters, and phenolic compounds. Depending on the plant species, environmental conditions, and extraction method employed, EOs may contain more than 300 individual constituents, many of which contribute to their characteristic aroma and broad spectrum of biological activities [62,63].

For decades, EOs and their bioactive constituents have been widely used because of their diverse biological activities, including antibacterial, antioxidant, anti-inflammatory, anticancer, allelopathic, repellent, and insecticidal properties [63]. In particular, their insecticidal activity has gained considerable attention for the control of mosquito vectors such as *Aedes aegypti*, *Anopheles* spp., and *Culex* spp., which are responsible for the transmission of major vector-borne diseases, including dengue, malaria, chikungunya, and Zika [62,64,65]. Likewise, in the veterinary field, EOs have been investigated as promising alternatives for the control of ectoparasites, particularly ticks, contributing to the development of more sustainable and environmentally friendly management strategies [66,67].

The insecticidal activity of EOs includes larvicidal, adulticidal, and repellent effects, as well as interference with key physiological processes such as feeding, oviposition, embryogenesis, and development [24,25,26,27]. These biological activities are closely associated with the chemical composition of EOs, whose variability and complex interactions among constituents play a fundamental role in determining their insecticidal efficacy [68,69]. However, it is difficult to attribute the observed effects solely to the major constituents, as in many cases the biological activity may result from synergistic interactions between dominant compounds and minor metabolites present within the mixture [28]. Therefore, it is important to evaluate both the whole EO and its individual constituents, since some isolated metabolites may exhibit high biological activity, whereas in other cases the overall efficacy of the oil depends on synergistic interactions among its components [70]. Additionally, plant-derived compounds represent promising alternatives due to their relatively low cost, reduced residual effects, and lower likelihood of inducing resistance compared with conventional synthetic insecticides [36,71,72].

### 5.1. Insecticidal Mechanisms of EOs

EOs exert their insecticidal activity through multiple mechanisms of action that affect essential physiological and biochemical processes in insects and other arthropods. Furthermore, the biological activity of EOs does not depend exclusively on their major constituents but also on synergistic interactions between major and minor metabolites, which can significantly enhance their insecticidal efficacy [69,73]. The principal mechanisms of action of EOs against insects are summarized in Table 1 [68,69,73,74].

### 5.2. EOs Against Flies That Cause Myiasis

EOs and their bioactive constituents have demonstrated a broad range of effects against myiasis-causing flies, affecting different stages of their life cycle as well as various essential physiological processes. Among the most frequently reported effects are larvicidal and adulticidal activities, together with repellent and oviposition-deterrent properties. Numerous studies have shown that EOs obtained from various plant species induce significant mortality in larvae of *Lucilia cuprina*, *Lucilia sericata*, *Cochliomyia macellaria*, *Cochliomyia hominivorax*, *Chrysomya bezziana*, *Chrysomya megacephala*, *Calliphora vomitoria*, and *Cephalopina titillator* [89,90,91,92]. A summary of the main biological effects reported for the evaluated EOs is presented in Table 2.

In addition to their direct insecticidal activity, several EOs interfere with key physiological processes associated with the development and reproduction of myiasis-causing flies. Oviposition inhibition and deterrent effects on adult females have been reported in *Lucilia sericata* and *Calliphora vomitoria*, significantly reducing egg deposition on treated substrates [93,94]. Other studies have demonstrated disruptions in embryogenesis and postembryonic development, including reduced egg hatchability, larval deformities, delayed pupation, and impaired adult development following the exposure of eggs or larvae to EOs from *Lantana camara* and other plant-derived metabolites [95].

Additionally, some EOs affect larval feeding behavior and digestive physiology through tissue damage and cytotoxic effects. For example, the EO of *Curcuma longa* and its major constituent, α-phellandrene, induced severe histological and ultrastructural alterations in the larval tissues of *Lucilia cuprina*, including cellular degeneration and damage to digestive structures, thereby compromising larval metabolism and survival [86]. Similar effects were observed following exposure to the EO of *Tagetes minuta*, whose caused tissue damage and cytotoxicity in exposed larvae [91].

Likewise, marked repellent and preventive effects against myiasis infestations have been reported. Certain EOs reduce the attraction and persistence of adult flies on wounds or organic substrates, thereby decreasing the risk of oviposition and larval establishment. In *Lucilia sericata*, EOs derived from vetiver, cinnamon, lavender, and other aromatic plant species demonstrated potential for preventing fly strike and reducing adult longevity [96,97]. Repellent activity and adult toxicity have also been documented in *Calliphora vomitoria* and *Chrysomya megacephala* following exposure to EOs rich in terpenes and phenylpropanoids [94,98,99].

**Table 2:** Essential oils evaluated against other species of flies that cause myiasis.

Plant Species	Major Components	Species	Test	Summary of Results	Ref
*Tagetes minuta*	Dihydrotagetone, trans-beta-ocimene, trans-tagetone	*Cochliomyia macellaria*	LCT	LC_50_ (24–48 h): ethanol = 0.678–0.580 μL cm^−2^. Mortality: 93.33% at 1.59 μL cm^−2^. Emergence inhibition: 87.27% at 0.7961 μL cm^−2^.	[90]
*Curcuma longa*	α-phellandrene, α-pinene, β-pinene	*Cochliomyia macellaria*	LCT	Mortality at 48 h at 1.27 μL cm^−2^: 96.66% (ethanol).	[100]
*Baccharis dracunculifolia*	*β*-pinene, D-limonene, *β*-nerolidol	*Cochliomyia macellaria*	LCT	LC_50_ (24–48 h): ethanol = 2.63–2.47 μL cm^−2^	[101]
*Clinopodium nubigenum*	Carvacrol, Pulegone	*Lucilia sericata*	ODT, ECT, ACT, FT, TAT	Oviposition deterrence: 100% at 0.8 μL cm^−2^. Toxicity in eggs: LC_50_ = 0.07 μL cm^−2^. Adults: LC_50_ = 0.278 μL insect^−1^.	[94]
*Lavandula angustifolia*	Linalool Linalyl acetate	*Lucilia sericata*	ODT, ECT, ACT	Deterrence: 100% at 0.8 μL cm^−2^ (3 h); 82.7% at 24 h. Toxicity in eggs: LC_50_ = 0.48 μL cm^−2^. Adults: LC_50_ = 0.393 μL insect^−1^.	[94]
*Chrysopogon zizanioides*	Not specified	*Lucilia sericata*	ODT, RT, ACT	At 0.2% → 100% mortality in 5 min. Strong oviposition deterrent effect.	[96]
*Cinnamomum zeylanicum*	Not specified	*Lucilia sericata*	ODT, RT, ACT	At 0.2% → 100% mortality in 5 min. Significant insecticidal and repellent activity.	[96]
*Lavandula angustifolia*	Not specified	*Lucilia sericata*	ODT, RT, ACT	At 0.2% → 100% mortality in 5 min. Repellent effect and reduction in longevity.	[96]
*Cinnamomum camphora*	Camphor, 1,8-cineole, safrole	*Lucilia sericata*	LIT	Mortality: 93.3% to 32%. It caused swelling and cuticular distortion.	[102]
*Lavandula angustifolia*	Linalool, linalyl acetate	*Lucilia sericata*	LIT	Mortality: 100% to 32%. Induced cuticular damage (swelling, deformation).	[102]
*Lactuca sativa*	Not specified	*Lucilia sericata*	LIT	LC_50_ = 0.57% (more potent). Marked decrease in pupation to 8%. Suppression of adult emergence to 2%.	[103]
*Matricaria chamomilla*	Not specified	*Lucilia sericata*	LIT	LC_50_ = 0.85%. Suppression of adult emergence at 2%.	[103]
*Pimpinella anisum*	Not specified	*Lucilia sericata*	LIT	LC_50_ = 2.74%. Lower relative toxicity. Induced morphological abnormalities.	[103]
*Rosmarinus officinalis*	Not specified	*Lucilia sericata*	LIT	LC_50_ = 6.77%. Induced deformities.	[103]
*Chrysopogon zizanioides*	Not specified	*Lucilia sericata*	LIT	High toxicity in L3: mortality = 93.33%. Negatively affected larval development.	[97]
*Cinnamomum zeylanicum*	Not specified	*Lucilia sericata*	LIT	Very high toxicity in L3: mortality = 95.56%. Significant effect on development.	[97]
*Curcuma longa*	*α*-phellandrene	*Lucilia cuprina*	LCT	High larvicidal activity and inhibition of adult emergence (96.22–100%).	[100]
*Tagetes minuta*	Dihydrotagetone, trans-ocimene, trans-tagetone	*Lucilia cuprina*	LCT	LC_50_ (24–48 h): acetone 1.02–0.73 μL cm^−2^; ethanol 3.37–1.75 μL cm^−2^; Tween 20 7.46–6.11 μL cm^−2^. Maximum mortality ~96.6% (48 h, acetone).	[91]
*Piper gaudichaudianum*	Germacrene B, δ-cadinene, γ-elemene	*Lucilia cuprina*	LCT	LC_50_ (24–48 h): ethanol = 3.69–2.19 μL cm^−2^; acetone = 9.14–6.05 μL cm^−2^.	[92]
*Piper betle*	Not specified	*Chrysomya bezziana*	LCT	4% induced 100% mortality in L1 and L2; 3% caused 100% mortality in L1 and 74% in L2; 2% resulted in 100% mortality of L1.	[89]
*Lippia sidoides*	Thymol	*Chrysomya megacephala*	LCT	Mortality ~90%. High insecticidal activity.	[99]
*Laurus nobilis*	1,8-cineole, linalool	*Chrysomya megacephala*	LCT	Mortality ~63.7%. Moderate activity.	[99]
*Lantana camara*	α-pinene, caryophyllene, geranyl acetate, eucalyptol	*Chrysomya megacephala*	ECT, PDT	High ovicidal activity, reduced pupation and adult emergence, with developmental abnormalities.	[95]
*Artemisia annua*	Artemisia ketone, 1,8-cineole	*Calliphora vomitoria*	ACT, FT	Contact toxicity: LC_50_ = 0.79 μL insect^−1^. Fumigation: LC_50_ = 88.09 μL L^−1^ of air.	[104]
*Artemisia dracunculus*	methyl chavicol, limonene	*Calliphora vomitoria*	ODT, ACT, FT	Oviposition: 100% inhibition at 0.05 μL cm^−2^. Contact toxicity: LC_50_ = 0.49 μL insect^−1^. Fumigation: LC_50_ = 49.55 μL L^−1^ of air.	[104]
*Allium sativum*	Allicin, diallyl disulfide, diallyl trisulfide	*Calliphora vomitoria*	EAG, ODT, ACT, FT	Strong repellent activity; complete inhibition of oviposition at ≥2.5 μL cm^−2^ for 24 h. Toxicity: LC_50_ = 0.44–1.97 μL insect^−1^; LC_50_ = 1.76–31.52 μL L^−1^.	[93]
*Rosmarinus officinalis*	1,8-cineole, camphor, α-pinene	*Calliphora vomitoria*	EAG, ODT, ACT, FT	Significant repellent activity; oviposition inhibition at ≥2.5 μL cm^−2^. Moderate toxicity in adults.	[93]
*Salvia officinalis*	Thujone, camphor, 1,8-cineole	*Calliphora vomitoria*	EAG, ODT, ACT, FT	Effective repellency and oviposition deterrence at ≥2.5 μL cm^−2^. Contact and fumigation toxicity.	[93]
*Origanum vulgare* (CC)	Carvacrol	*Calliphora vomitoria*	ACT, OCT	Toxicity in adults: LC_50_ = 0.14–0.31 μL insect^−1^. Toxicity in eggs: LC_50_ = 0.008–0.038 μL cm^−2^.	[98]
*Origanum vulgare* (TCC)	Thymol, *p*-cymene	*Calliphora vomitoria*	ACT, OCT	Toxicity in adults: LC_50_ = 0.14–0.31 μL insect^−1^. Ovicidal: LC_50_ = 0.008–0.038 μL cm^−2^.	[98]
*Origanum vulgare* (TTC)	Thymol, γ-terpinene	*Calliphora vomitoria*	ACT, OCT	Toxicity in adults and eggs within reported ranges: LC_50_ = 0.14–0.31 μL insect^−1^, LC_50_ = 0.008–0.038 μL cm^−2^.	[98]
*Cucurbita maxima*	Not specified	*Cephalopina titillator*	LIT	100% mortality at 2% (24 h). LC_50_ = 0.20%. Total inhibition of pupation and emergence.	[105]
*Lupinus luteus*	Not specified	*Cephalopina titillator*	LIT	100% mortality to 30%. LC_50_ = 0.47%.	[105]
*Allium sativum*	Not specified	*Cephalopina titillator*	LIT	100% mortality at 7.5%. LC_50_ = 0.44%.	[105]
*Mentha piperita*	Not specified	*Cephalopina titillator*	LIT	100% mortality at 7.5%. LC_50_ = 0.42%. Greater incidence of deformities: 44% larvae (7.5%) and 40% pupae (2%).	[105]

LIT, Larval Immersion Test; LCT, Larval Contact Test; ODT, Oviposition Deterrence Test; ECT, Egg Contact Test; ACT, Adult Contact Test; FT, Fumigant Test; TAT, Topical Application Test; RT, Repellency Test; PDT, Postembryonic Development Test; EAG, Electroantennography; L1, first-instar larva; L2, second-instar larva; L3, third-instar larva; LC_50_, median lethal concentration; h, hours; CC, carvacrol chemotype; TCC, thymol/p-cymene chemotype; TTC, thymol/γ-terpinene chemotype.

Collectively, these findings indicate that EOs exhibit multifactorial mechanisms of action against myiasis-causing flies. In addition to their larvicidal and adulticidal activities, they interfere with critical physiological processes associated with feeding, reproduction, embryogenesis, and development, highlighting their potential as promising alternatives for integrated and sustainable myiasis control strategies.

### 5.3. Effects of EOs and Their Components Against C. hominivorax

Currently, the search for sustainable alternatives for the control of *C. hominivorax*, the primary causative agent of traumatic myiasis in domestic animals and humans throughout Latin America, is an area of growing interest. In this context, EOs have emerged as promising candidates for the development of novel control strategies based on natural compounds. However, studies specifically focused on the evaluation of EOs against *C. hominivorax* remain limited. Among the most relevant investigations is the study conducted by Medeiros et al. [70], who evaluated the in vitro larvicidal activity of 15 EOs against third instar (L3) larvae of *C. hominivorax* (Table 3). The evaluated EOs were obtained from plant species including *Thymus vulgaris*, *Origanum vulgare*, *Illicium verum*, *Lavandula hybrida*, *Rosmarinus officinalis*, *Cymbopogon winterianus*, and *Cinnamomum cassia*, among others. Larvicidal activity was evaluated using filter paper contact bioassays in which EO solutions, prepared at concentrations ranging from 10,000 to 100,000 μg mL^−1^, were applied to filter paper discs. Larval mortality was assessed by microscopic examination, considering morphological alterations and the absence of movement. Because the bioassay was based on the deposition of the compounds on a defined filter paper surface, larvicidal activity was expressed as LC_50_ and LC_90_ values in μg cm^−2^, representing the amount of active compound deposited per unit area. The median lethal concentration (LC_50_) and the 90% lethal concentration (LC_90_) were subsequently estimated by Probit analysis.

**Table 3:** Essential oils and components evaluated against *Cochliomyia hominivorax* larvae.

Treatments	Plant Species	Major Components	Summary of Results	Ref.
Essential oils	*Salvia sclarea*	Linalyl acetate, linalool	Mortality of 3.3% at 25,000 μg mL^−1^	[70]
	*Rosmarinus officinalis*	Eucalyptol, Camphor	Mortality of 33.6% at 100,000 μg mL^−1^	
	*Lavandula hybrida*	Linalool, Linalyl acetate	Mortality of 16.7% at 50,000 μg mL^−1^	
	*Citrus bergamia*	Linalyl acetate, limonene	Mortality of 43.3% at 100,000 μg mL^−1^	
	*Citrus paradisi*	Linalyl acetate	Mortality of 38.4% at 100,000 μg mL^−1^	
	*Juniperus virginiana*	α-cedrene, cedrol	Mortality of 6.7% at 50,000 μg mL^−1^	
	*Copaifera reticulata*	β-caryophyllene	Mortality of 20.0% at 50,000 μg mL^−1^	
	*Cymbopogon flexuosus*	Geranial, geraniol	Mortality of 46.7% at 100,000 μg mL^−1^	
	*Eugenia caryophyllus*	Eugenol	Mortality of 53.3% at 100,000 μg mL^−1^	
	*Cinnamomum cassia*	Cinnamaldehyde	Mortality of 53.3% at 100,000 μg mL^−1^	
	*Pelargonium roseum*	Citronelol, geraniol	Mortality of 23.3% at 75,000 μg mL^−1^	
	*Cymbopogon winterianus*	Citronellal, geraniol	Mortality of 33.3% at 75,000 μg mL^−1^	
	*Illicium verum*	(E)-anethole	100% mortality at 100,000 μg mL^−1^. LC_50_: 418.1 µg cm^−2^ (48 h)	
	*Thymus vulgaris*	Thymol, Ocimene	100% mortality at 50,000 μg mL^−1^. LC_50_: 407.1 µg/cm^2^ (24 h) and 314.2 µg cm^−2^ (48 h)	
	*Origanum vulgare*	Carvacrol	100% mortality at 75,000 μg mL^−1^. LC_50_: 540.9 µg/cm^2^ (24 h) and 253.8 µg cm^−2^ (48 h)	
Components	-	Trans-anethole	100% mortality at 100,000 μg mL^−1^. LC_50_: 559.4 µg cm^−2^ (48 h)	
	-	Thymol	Mortality of 98.3% at 80,000 μg mL^−1^. LC_50_: 255.6 µg/cm^2^ (24 h) and 102.3 µg cm^−2^ (48 h)	
	-	Carvacrol	95% mortality at 100,000 μg mL^−1^. LC_50_: 970.5 µg/cm^2^ (24 h) and 931.1 µg cm^−2^ (48 h)	

LC_50_: Lethal concentration of 50%.

The results demonstrated that the EOs of *T. vulgaris*, *O. vulgare*, and *I. verum* were the only oils capable of inducing 100% mortality in L3 larvae of *C. hominivorax* at one or more of the tested concentrations. Among them, *T. vulgaris* EO exhibited the highest larvicidal activity, achieving 100% mortality even after 24 h of exposure and at lower concentrations than those required for the other EOs evaluated. In contrast, most of the remaining EOs displayed limited activity, generally producing mortality rates below 55% [70].

Chemical analysis revealed that the EO of *T. vulgaris* contained thymol as its major constituent (44.7%), together with ocimene (26.6%), whereas *O. vulgare* was characterized by a high carvacrol content (76.2%), and *I. verum* contained trans-anethole as its predominant compound (80%). These major constituents were subsequently evaluated individually to determine their contribution to the observed larvicidal activity. Thymol was identified as the most active compound, exhibiting LC_50_ values of 255.6 and 102.3 μg cm^−2^ after 24 and 48 h of exposure, respectively, which were lower than those obtained for the whole *T. vulgaris* EO (LC_50_ = 407.1 and 314.2 μg cm^−2^) [70]. These findings indicate that thymol is the primary contributor to the insecticidal activity of *T. vulgaris*. The greater potency of the isolated compound suggests that certain secondary constituents, such as monoterpene hydrocarbons (e.g., ocimene), may contribute little to insecticidal activity and could even reduce the overall biological efficacy through dilution effects or weak antagonistic interactions.

In contrast, the EO of *O. vulgare* exhibited greater larvicidal activity than its major constituent, carvacrol, suggesting that the insecticidal efficacy of this EO results from synergistic interactions among its constituents [70]. This observation indicates that the combination of metabolites present in the whole EO may enhance the biological activity of carvacrol, thereby increasing its toxicity against larvae. Similarly, the greater efficacy of *I. verum* EO compared with isolated trans-anethole may be attributed to synergistic interactions between this compound and other minor metabolites present in the EO, although these mechanisms remain to be experimentally confirmed [70].

Collectively, these findings demonstrate that the insecticidal activity of EOs against *C. hominivorax* depends not only on their major constituents but also on the overall chemical composition of the EO and the interactions among major and minor metabolites, which may be either synergistic or antagonistic depending on the chemical profile of each EO. These observations underscore the importance of evaluating both whole EOs and their isolated constituents to gain a better understanding of their mechanisms of action and to optimize the development of plant-derived insecticides for the control of myiasis-causing flies.

### 5.4. Advantages and Limitations of Essential Oils as Insecticides

**Figure 3:**
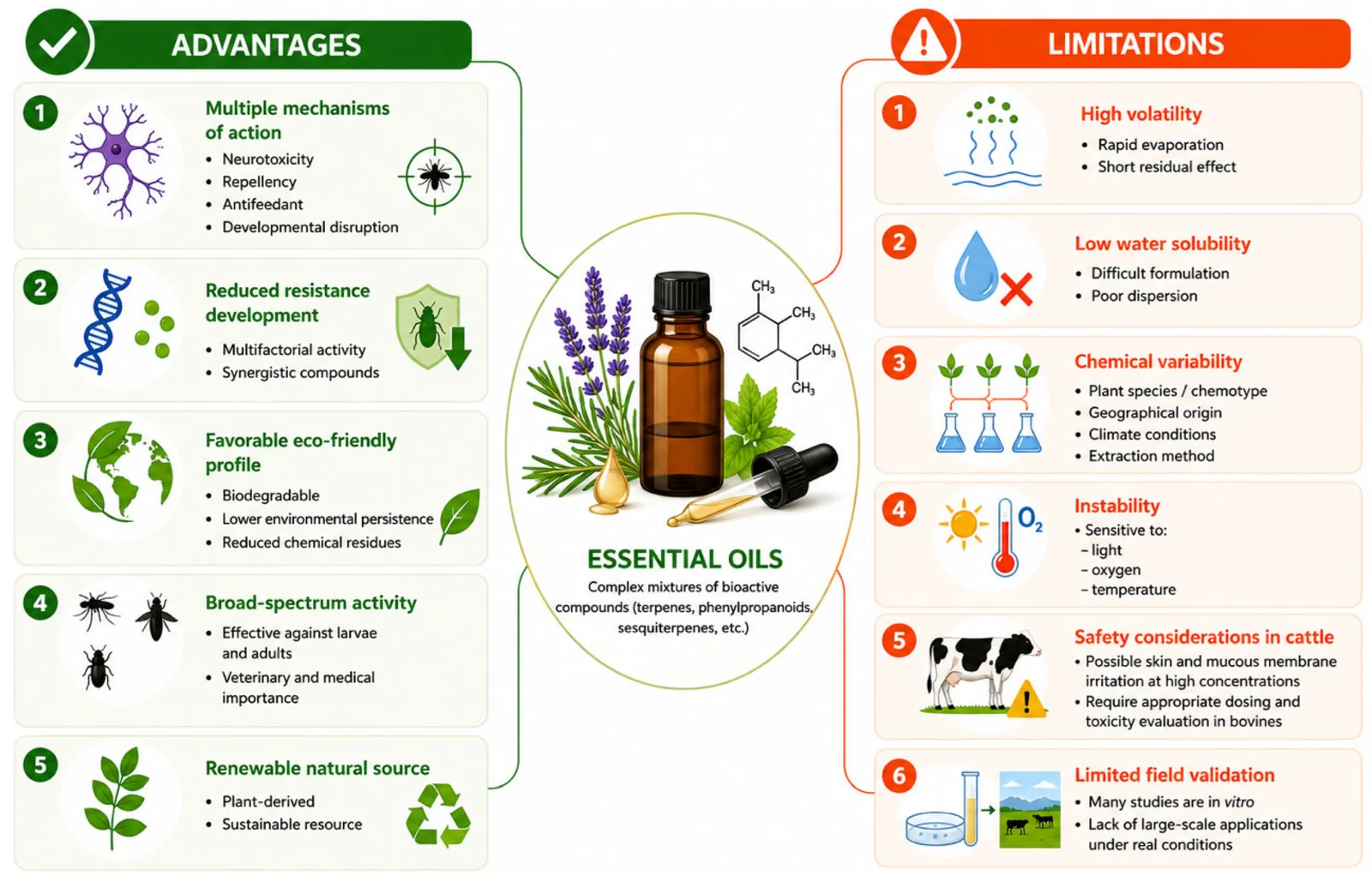
Advantages and limitations of essential oils as natural insecticides.

EOs have attracted increasing attention as natural insecticides due to their diverse array of bioactive compounds and multiple mechanisms of action. The main advantages and limitations of EOs as natural insecticides are summarized in Figure 3, including their biological activity, mechanisms of action, stability, and applicability in insect control strategies [106,107,108,109].

## 6. Nano-Enabled Plant-Derived Insect Control Strategies

### 6.1. Nanocarrier Systems for EOs

Nanotechnology is an interdisciplinary field dedicated to the design and development of nanoscale materials and systems whose unique physicochemical properties have enabled significant advances in biological, chemical, and technological applications [36,110]. In nanocarrier systems, these properties are closely associated with their nanoscale dimensions, which increase the surface-area-to-volume ratio and promote more efficient interactions with biological tissues, cellular membranes, and target organisms [111,112,113]. This feature is particularly relevant for insecticidal applications, as it facilitates the penetration of bioactive compounds through protective barriers such as the insect cuticle, thereby enhancing their bioavailability and biological efficacy [36,47].

**Figure 4:**
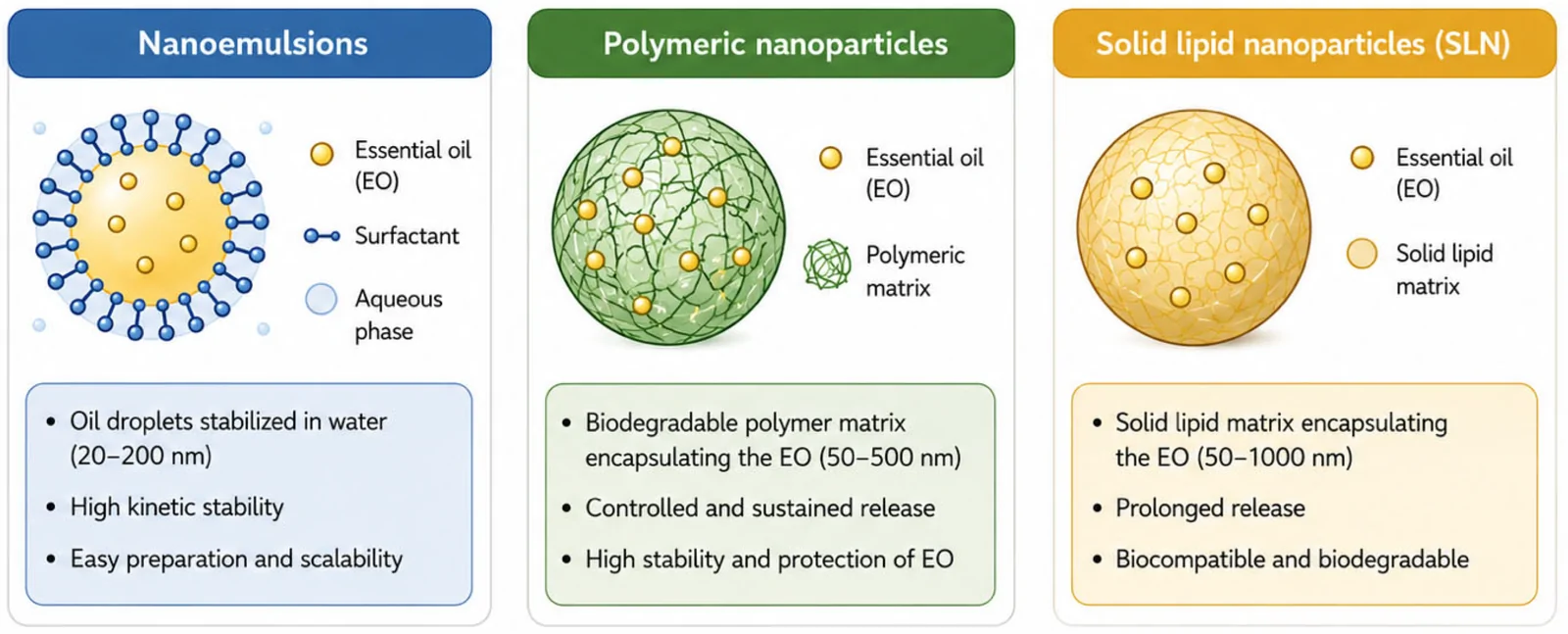
Nanodelivery systems of essential oils and their advantages for insecticidal applications.

Among the main nanocarriers investigated for the delivery of insecticidal compounds are polymeric nanoparticles, lipid nanoparticles, and nanoemulsions. The general characteristics of these nanodelivery systems are summarized in Figure 4 [40,114,115,116,117,118]. These nanoscale platforms enable the encapsulation of both hydrophilic and hydrophobic bioactive compounds, improving their stability, dispersion, and bioavailability [36,47].

The enhanced efficacy of plant-derived compounds through nanotechnology can largely be attributed to the ability of nanocarriers to protect active compounds from environmental factors such as light, oxygen, humidity, and temperature, which may induce degradation, oxidation, or volatilization processes, particularly in EOs rich in volatile constituents [119,120,121]. Consequently, nanocarriers help preserve the biological activity of these compounds until they reach the target organism [36,120,122].

Furthermore, these systems can provide controlled and sustained release profiles, maintaining effective concentrations of the active ingredient over extended periods [120,121]. This not only improves biological efficacy but may also reduce environmental exposure and minimize potential toxic effects associated with high doses or repeated applications [123,124,125]. In addition, certain nanocarriers can be engineered with bioadhesive or mucoadhesive properties, promoting prolonged retention on biological tissues or wounded surfaces, which may represent an important advantage for myiasis treatment applications [126,127,128].

Moreover, nanocarriers possess several characteristics that can significantly improve the performance of natural insecticidal compounds, including enhanced compatibility with aqueous media, improved physicochemical stability, increased intracellular penetration, and the reduction or elimination of potentially toxic solvents and diluents [129,130,131]. Their versatile structural design, which allows control over particle size, morphology, surface charge, and release mechanisms, enables the optimization of interactions with tissues, cells, and target organisms, thereby maximizing biological efficacy of nanoencapsulated formulations [132].

### 6.2. Effects on Flies That Cause Myiasis and Other Insects

The incorporation of plant-derived bioactive compounds, particularly EOs, into nanocarrier systems has emerged as a promising strategy to enhance their applicability for the control of myiasis-causing flies. Although studies specifically evaluating nanoformulations against *C. hominivorax* remain limited, growing evidence supports the efficacy of nanoencapsulated botanical compounds against this species and other dipterans of veterinary and public health importance.

Among the few studies available for *C. hominivorax*, the evaluation of a *Cymbopogon winterianus* EO nanoemulsion is particularly noteworthy [133]. This commercial oil-in-water nanoemulsion containing 15% EO exhibited remarkable insecticidal activity, achieving 100% mortality in eggs and first- and second-instar larvae, while mortality in third-instar larvae reached 69.3%. Additionally, the formulation significantly inhibited oviposition by adult females, demonstrating not only larvicidal activity but also ovicidal and reproductive effects [133]. The reduced efficacy observed against third-instar larvae is likely associated with their more advanced development, including a thicker and more sclerotized cuticle, larger body size, and deeper penetration into host tissues, which may limit the penetration and diffusion of bioactive compounds. Although this finding highlights the challenges associated with controlling advanced larval stages, it also underscores the need to further optimize nanoformulation properties to enhance bioactive delivery. Overall, these results suggest that nanoemulsified *C. winterianus* EO represents a promising botanical alternative for the treatment and prevention of myiasis caused by *C. hominivorax*.

Similarly, a nanoemulsion formulated with *Baccharis dracunculifolia* EO was evaluated against *C. hominivorax* larvae [134]. The formulation produced concentration-dependent mortality, reaching up to 97% larval mortality at a concentration of 15% (*w*/*v*). Importantly, the nanoemulsion maintained high insecticidal activity even after 120 days of storage, achieving mortality rates of up to 92% [134]. These results highlight the potential of nanoemulsions to overcome the inherent instability of many EOs without compromising their larvicidal efficacy.

Studies conducted on other dipteran species of veterinary importance also support the use of nano-insecticides. Nanoemulsions formulated with *Cymbopogon citratus* EO exhibited enhanced insecticidal activity against adults of *Lucilia cuprina*, one of the principal species associated with ovine flystrike [135]. Compared with the free EO, nanoemulsification increased insect mortality in both topical application bioassays and impregnated-paper exposure tests, demonstrating that nanostructured systems can improve the bioavailability and efficacy of volatile botanical compounds [135]. Similar results were observed against *Musca domestica*, suggesting a broad spectrum of activity against different dipteran species [135].

Likewise, nanoemulsions prepared with *Pelargonium graveolens* EO exhibited superior insecticidal activity against *M. domestica*. The nanoemulsified formulation reduced larval LC_50_ values from 4.29% to 1.50%, indicating a substantial increase in insecticidal potency [136]. Furthermore, complete inhibition of adult emergence was observed in treated pupae. Interestingly, combining the EO with sesame oil further enhanced insecticidal activity, highlighting the importance of formulation strategies for optimizing biological efficacy [136].

Other studies have investigated nanostructured formulations of eucalyptus (*Eucalyptus globulus*) and cinnamon (*Cinnamomum zeylanicum*) EOs against adult flies [137]. *Eucalyptus* EO incorporated into poly(ε-caprolactone) nanoparticles significantly reduced *M. domestica* populations and exhibited pronounced repellent activity against the horn fly, *Haematobia irritans*, reducing infestations in naturally infested cattle by up to 66.6% after 24 h. Similarly, a cinnamon EO nanoemulsion achieved 100% mortality of *M. domestica* adults after 90 min of exposure and significantly reduced *H. irritans* populations under field conditions [137]. These findings indicate that nanoformulations can simultaneously provide insecticidal and repellent effects, two highly desirable characteristics for integrated fly management programs.

In addition to nanoemulsions, polymeric nanoparticles have demonstrated considerable potential for enhancing the efficacy of plant-derived bioactive compounds against insects. For example, poly(ε-caprolactone) nanoparticles loaded with *Rosmarinus officinalis* EO exhibited prolonged insecticidal activity against adults of *Drosophila suzukii* [138]. Although both the free oil and the nanoencapsulated formulation showed similar toxicity (LD_50_ ≈ 9.1 g L^−1^), nanoencapsulation enhanced and extended the insecticidal effect over time [138]. Similarly, several bioactive terpenes and phenylpropanoids, including carvacrol, L-(-)-carvone, trans-anethole, and cinnamaldehyde, were incorporated into poly(ε-caprolactone) nanoparticles and evaluated against *D. suzukii* adults [139]. The nanoencapsulated formulations exhibited more prolonged insecticidal activity than the corresponding pure compounds, with carvacrol-loaded nanoparticles showing particularly promising results. In addition, physiological alterations associated with oxidative stress and histopathological changes in muscles, the midgut, and the fat body were observed in treated insects [139]. Importantly, these nanoformulations did not produce significant adverse effects on the beneficial parasitoid *Palmistichus elaeisis*, indicating a degree of selectivity toward non-target organisms [139].

Collectively, these studies demonstrate that nanocarrier systems can substantially enhance the efficacy of plant-derived bioactive compounds against myiasis-causing flies and other dipterans. The main improvements reported include increased larvicidal, ovicidal, adulticidal, and repellent activities, enhanced storage stability, reduced effective concentrations, and improved capacity to interfere with key physiological processes related to insect development, reproduction, oviposition, and nervous system function. Although research specifically focused on *C. hominivorax* remains limited, the available evidence strongly supports the development of nanotechnology-based botanical formulations as promising and sustainable tools for the prevention and control of myiasis.

### 6.3. Innovative Approaches for Managing C. hominivorax

The evidence summarized in this review indicates that nanocarrier-based delivery systems represent a promising strategy to enhance the practical application of EOs for the management of *C. hominivorax* myiasis. By improving the stability, retention, and local delivery of bioactive compounds, these systems may overcome several limitations associated with free EOs, including their volatility, physicochemical instability, and poor aqueous dispersibility [40,114,115,116,117,118]. Such characteristics are particularly advantageous for topical treatment of myiasis wounds, where prolonged contact with larvae and damaged tissues is essential to maximize biological efficacy [140,141,142].

Among the botanical compounds reviewed, the EOs of *I. verum*, *T. vulgaris*, and *O. vulgare*, together with their principal constituents trans-anethole, thymol, and carvacrol, represent promising candidates for incorporation into nanocarrier systems due to their demonstrated larvicidal activity against *C. hominivorax* [70]. Beyond improving insecticidal efficacy, nanoencapsulation may further harness other biological properties of these compounds, including antimicrobial, anti-inflammatory, antioxidant, and wound-healing activities [143,144,145,146]. These multifunctional effects are particularly relevant because myiasis lesions are characterized by extensive tissue destruction, inflammation, and a high risk of secondary bacterial infections. Consequently, nanoformulated EOs may simultaneously promote parasite elimination, reduce microbial burden, and support tissue repair [147,148].

Despite these encouraging findings, several scientific, technical, economic, and regulatory challenges must still be addressed before nanotechnology-based botanical formulations can be translated into veterinary practice. Future research should prioritize the identification and chemical standardization of the most active constituents of EOs, optimization of nanocarrier formulations, evaluation of tissue biocompatibility and local safety, and the establishment of reproducible and cost-effective large-scale manufacturing processes [149,150]. Likewise, comprehensive in vivo studies and large-scale field trials under commercial livestock conditions are required to validate efficacy, determine optimal dosing regimens, and assess long-term performance under practical conditions [150]. In parallel, the development of standardized quality control methods and adequate physicochemical characterization will be essential to ensure the consistency and reliability of nanoparticle-based veterinary products. Furthermore, regulatory frameworks specifically adapted to these formulations will be crucial to facilitate their commercialization and successful translation into practical veterinary applications [36,125,151].

## 7. Conclusions

EOs represent promising natural alternatives for the control of *C. hominivorax* due to their larvicidal, ovicidal, adulticidal, and repellent activities, as well as their ability to interfere with essential physiological processes of the insect. Although the information available for this species remains limited, several EOs and their major bioactive constituents have demonstrated significant insecticidal activity. In particular, the EOs of *Illicium verum*, *Thymus vulgaris*, and *Origanum vulgare*, together with their major constituents trans-anethole, carvacrol, and especially thymol, stand out as promising candidates for incorporation into nanocarrier systems aimed at myiasis control.

The nanoencapsulation of these compounds could improve their stability, bioavailability, and biological efficacy, while promoting the development of multifunctional formulations capable of combining insecticidal activity against *C. hominivorax* with antimicrobial, anti-inflammatory, and wound-healing properties. Consequently, such formulations may contribute not only to parasite control but also to the prevention of secondary infections and the recovery of tissues damaged by larval infestation.

Overall, the integration of EOs with nanocarrier systems represents a promising and environmentally sustainable strategy for the prevention and management of *C. hominivorax* myiasis. Continued advances in formulation science, veterinary pharmacology, and translational research are expected to accelerate the development of safe, effective, and commercially viable nanotechnology-based alternatives. However, further studies focused specifically on *C. hominivorax* are required to validate these approaches under field conditions, elucidate their mechanisms of action, and confirm their efficacy and safety in real-world applications against this parasite of major economic and veterinary importance.

## Figures and Tables

**Table 1:** Biological mechanisms of action of essential oils against insects.

Mechanism	Overview	Biological Effects	Physiological Targets	Ref
Neurotoxicity	Disruption of nerve transmission through interactions with neurotransmitters and ion channels	Hyperactivity, incoordination, paralysis, and death	Acetylcholinesterase, octopamine receptors, and GABA receptors.	[68,73,75]
Alteration of cell membranes	Disruption of the integrity and permeability of cell membranes	Loss of homeostasis, leakage of cellular contents, and necrosis	Plasma membranes and cell organelles	[69,76]
Oxidative stress	Excessive production of reactive oxygen species	Oxidative damage, apoptosis, and tissue degeneration	Cellular antioxidant systems	[73,76,77]
Repellency	Alteration of the insect’s chemical perception and orientation	Avoidance of host approach, feeding, and oviposition.	Olfactory and sensory receptors	[78,79,80]
Antifeeding activity	Inhibition or reduction in feeding	Reduced food intake and stunted growth	Gustatory receptors and the digestive system	[80,81]
Inhibition of oviposition	Alteration of chemical signals related to reproduction	Reduced oviposition	Reproductive system and ovipositor behavior	[82,83]
Interference with development and metamorphosis	Alteration of hormonal and growth processes	Disruption of molting, pupation, and adult emergence	Juvenile hormone and ecdysone	[84,85]
Cuticle alteration and dehydration	Dissolution or alteration of cuticular lipids	Water loss and reduced barrier protection	Cuticle and lipid layer	[86,87,88]

GABA: gamma-aminobutyric acid.

## Data Availability

Data is contained within the article.
